# Application of bedside ultrasound monitoring gastric residual volume in early enteral nutrition of ventilation patients with acute exacerbation of chronic obstructive pulmonary disease

**DOI:** 10.3389/fmed.2026.1847620

**Published:** 2026-06-11

**Authors:** Yanfei Yu, Hongmei Zhou, Lingsha Wu

**Affiliations:** The Second Hospital of Jiaxing, Jiaxing, China

**Keywords:** acute exacerbation of chronic obstructive pulmonary disease, enteral nutrition, gastric residual volume, mechanical ventilation, ultrasonography

## Abstract

**Objective:**

To explore the clinical utility of bedside ultrasound for monitoring gastric residual volume in early enteral nutrition among mechanically ventilated patients with acute exacerbation of chronic obstructive pulmonary disease (AECOPD).

**Methods:**

A total of 60 patients with AECOPD and undergoing mechanical ventilation were selected from the intensive care unit (ICU) of the Second Hospital of Jiaxing between April 2020 and April 2023. They were randomly assigned to either an ultrasound group or a control group, with each group consisting of 30 cases. In the ultrasound group, gastric residual volume was monitored using bedside ultrasound measurement of gastric antrum area, while in the control group it was monitored using gastric fluid withdrawal method. The monitoring frequency was set at once every 4 hours, and enteral nutrition was dynamically adjusted based on the monitoring results. The operation time of gastric residual volume monitoring, standard rate of enteral nutrition within 48 h, standard time of enteral nutrition, and the incidence of adverse reactions were compared between the two groups.

**Results:**

The operation time for gastric residual monitoring and the duration required for enteral nutrition to reach the standard were significantly shorter in the ultrasound group compared to the control group (*p* < 0.05). Additionally, the incidence of diarrhea, constipation, abdominal distension, and aspiration in the ultrasound group was significantly lower compared to the control group (*p* < 0.05). However, the rate of 48-h enteral nutrition compliance did not exhibit a statistically significant difference between the two groups (*p* > 0.05).

**Conclusion:**

The utilization of bedside ultrasound for monitoring gastric residual volume is both convenient and accurate, thereby facilitating early enteral nutrition in patients with AECOPD and mechanical ventilation, while concurrently reducing the occurrence of complications associated with feeding intolerance.

## 1 Introduction

Chronic obstructive pulmonary disease (COPD) ranks as the third leading cause of mortality in China, posing a significant threat to public health and social progress due to its elevated incidence, mortality rate, disability burden, and substantial healthcare expenses (1). The malnutrition-induced damage to muscle structure and function can lead to a reduction in respiratory muscle reserve capacity and exacerbate disease progression, thereby serving as an independent risk factor for the unfavorable prognosis of patients with COPD (2). Mechanical ventilation is a commonly employed therapy for acute exacerbation of chronic obstructive pulmonary disease (AECOPD). However, positive pressure ventilation during mechanical ventilation may elevate intra-abdominal pressure and impede intestinal function, while the administration of analgesia and sedation in conjunction with mechanical ventilation can hinder gastrointestinal peristalsis (3). Consequently, this leads to an elevation in gastric residual volume (GRV) and the subsequent development of aspiration pneumonia, feeding intolerance, and other complications during the initial enteral nutrition process in patients with AECOPD. Therefore, continuous monitoring of GRV is imperative during enteral nutrition administration. Although the gastric fluid withdrawal method is frequently employed in nursing practice for this purpose, its accuracy remains a subject of controversy. The objective of this study is to investigate the potential value of bedside ultrasound in monitoring GRV for facilitating early enteral nutrition implementation in patients with AECOPD and mechanical ventilation.

## 2 Information and methods

### 2.1 Clinical information

The study included 60 patients admitted to the ICU at the Second Hospital of Jiaxing between April 2021 and April 2023 for mechanical ventilation via endotracheal intubation due to AECOPD, who were scheduled to receive early enteral nutrition via nasogastric tube. The sample size calculation formula was: n1 = n2 = 2(Z_α/2_ + Z_β_)*σ*/*δ* 2. Based on the results of the pilot study, Z_α/2_, Z_β_, σ, and δ were determined to be 1.96, 1.28, 4.45, and 4.13, respectively. Substituting these values into the formula yielded a required sample size of 27 per group. Taking into account a 10% attrition rate and dropout rate, the sample size for each group was set at 30, resulting in a total of 60 patients included in the study. Inclusion criteria: ① AECOPD diagnosis criteria should adhere to relevant guidelines (4); ② Participants aged between 18 and 75 years old are eligible; ③ Initiation of enteral nutrition within the first 24 h of mechanical ventilation is required. Exclusion criteria: ① Hemodynamic instability; ② Excessive obesity or severe abdominal distension that precludes ultrasound examination; ③ Previous history of abdominal surgery resulting in gastrointestinal structural abnormalities or patients with severe abdominal infection; ④ Patients with aspiration pneumonia, diarrhea, abdominal distension, gastrointestinal bleeding, or other contraindications to enteral nutrition prior to admission into the ICU. Dropout criteria: ① Patients were discharged voluntarily, deceased, or requested to withdraw from the study during the designated period; ② Enteral nutrition was discontinued for over 12 h due to exacerbation of the primary ailment, severe secondary complications, or other justifiable reasons throughout the investigation. In this study, subjects were assigned to groups using a random number table. Based on the predetermined sample size, the starting row and column positions were randomly selected from the random number table. Numbers were read sequentially from left to right and top to bottom, and each subject meeting the inclusion criteria was assigned a unique identification number. The resulting random numbers were arranged in ascending order, and according to the predetermined grouping scheme, subjects with odd-numbered positions were assigned to the experimental group, while those with even-numbered positions were assigned to the control group. Additionally, to avoid selection bias, allocation was concealed throughout the study: a dedicated individual, who was not involved in patient enrollment or group assignment, independently generated the random sequence and prepared the group assignment cards, which were sealed in sequentially numbered, opaque envelopes. Upon enrollment, participants were assigned to groups by sequentially opening the envelopes in the order of recruitment. The person performing the group assignment was unaware of the random allocation sequence in advance, effectively eliminating human interference and predetermination of group assignment. There were no statistically significant differences in the general characteristics between the two groups (*p* > 0.05), indicating their comparability, as presented in Table 1. The present study was granted approval by the Medical Ethics Committee of our institution, and informed consent was obtained from either the patient or their designated family member.

**Table 1:** Comparison of general data of patients between the two groups.

Item	Ultrasound group (*n* = 30)	Control group (*n* = 30)	t/Z/χ2	OR (95%CI)	*P*
Gender/*n* (%)			0.069	0.90 (−0.33, 2.44)	0.793
Men	17	18			
Women	13	12			
Age (year, $\bar{x}$ ±*s*)	59.4 ± 15.3	58.6 ± 13.7	0.768	0.82 (−6.62, 8.27)	0.449
BMI (kg/m^2^, $\bar{x}$ ±*s*)	22.6 ± 3.1	22.9 ± 2.8	0.512	−0.34 (−1.86, 1.22)	0.613
Time to start enteral nutrition [h, M (Q1, Q3)]	16 (7, 21)	14 (9, 23)	0.752	2.40 (−4.05, 6.02)	0.452
APACHE II score (score, $\bar{x}$ ±*s*)	16.2 ± 2.8	15.8 ± 2.6	0.417	0.45 (−1.24, 1.83)	0.680
SOFA score (score, $\bar{x}$ ±*s*)	3.6 ± 0.5	3.5 ± 0.4	0.174	0.11 (−0.13, 0.34)	0.863
Diabetes			0.267	1.31 (0.48, 3.57)	0.606
Yes	16	14			
No	14	16			
High blood pressure			0.341	0.74 (0.24, 2.31)	0.559
Yes	21	23			
No	9	7			

BMI, Body Mass Index; APACHE II Score, Acute Physiology and Chronic Health Evaluation; SOFA Score, Sequential Organ Failure Assessment.

### 2.2 Methods

#### 2.2.1 Enteral nutrition regimen

The initiation of enteral nutrition support via nasogastric tube was considered if the patient’s mechanical ventilation exceeded 12 h without any relevant contraindications. The formulation of the enteral nutrition scheme followed the Guidelines for the Implementation and Evaluation of Nutritional Support Treatment for Adult Critically Ill Patients published in 2016 (2). Nengquanli (an enteral nutrition suspension manufactured by Nutricia Company) was selected as the preferred enteral nutrition preparation, and continuous nasal feeding was administered for a duration of 24 h using a nutritional pump, with a target value set at 25 ~ 30 kcal/(kg·d). During enteral nutrition, the head of the bed was elevated at an angle of 30–45°, oral hygiene measures were performed thrice daily, infusion tubes for nutrient solution were replaced on a daily basis, monitoring of water and electrolyte changes was conducted, and blood glucose levels were controlled to not exceed 10 mmol/L. The GRV was monitored at 4-h intervals. Enteral nutrition administration was halted if the GRV exceeded 500 mL, adjusted or maintained at the original rate if the GRV ranged between 200 and 500 mL, and continued or escalated to reach the expected rate if the GRV fell below 200 mL.

#### 2.2.2 Monitoring of GRV

##### 2.2.2.1 Control group

The GRV was monitored via gastric juice aspiration. The administration of nasal feeding pump was temporarily halted and the timer was initiated. Gastric juice was aspirated through the nasogastric tube using a 50 mL syringe, collected in a disposable sterile dressing bowl, and the total volume of gastric juice was recorded. The relevant operations were conducted by experienced nurses who underwent standardization training prior to the commencement of the study.

##### 2.2.2.2 Ultrasound group

The GRV was monitored using ultrasound. The patient stopped nasogastric feeding and the timing began, assuming the supine position on the right side while employing an ultrasound probe (Italy Yum Mylab Gold25 ultrasound machine, CA430 probe). This allowed for visualization of a single section of the gastric antrum via a vertical abdominal scan beneath the xiphoid process, with subsequent measurement of its cross-sectional area. According to the relevant literature (5), the calculation formula for GRV was as follows: GRV (mL) = 27.0 + 14.6 * gastric antral area (cm^2^) − 1.28 * age (years). After three measurements, the average value was obtained, and subsequently, the patient returned to their original position for monitoring while adjusting the pump injection speed based on GRV. All bedside ultrasound technicians are qualified, highly skilled nurses who have completed the specialized training in critical care ultrasound required by the Jiangsu Provincial Society of Critical Care Medicine. There are six technicians in total. Each bedside ultrasound evaluation is performed by two qualified nurses working together; in the event of a discrepancy, a third specialized nurse will conduct the evaluation.

### 2.3 Evaluation indicators

The standard amount of early enteral nutrition was taken as the end point of observation. The primary outcome measures were as follows: ① Operation time: The actual time required for each GRV assessment was recorded for patients in both groups from the start of enteral feeding to 48 h post-initiation; ② The rate of achieving target enteral nutrition levels within 48 h of feeding and the time to achieve these levels, with the target considered met when the actual enteral nutrition intake reached 70% of the target value. The secondary outcome measures were: adverse events such as ventilator-associated pneumonia (VAP) and feeding intolerance: diarrhea (this refers to a bowel movement frequency of more than 3 times per day, with loose stools that are watery and may contain mucus, pus, blood, and undigested food.), constipation (symptoms such as a decrease in the frequency of bowel movements, reduced stool volume, hard stools, and straining during bowel movements), bloating (this abdominal discomfort and feeling of bloating are caused by slowed intestinal motility and gas in the gastrointestinal tract.), gastrointestinal bleeding (this refers to bleeding occurring between the esophagus and the anus, which is classified as upper gastrointestinal bleeding and lower gastrointestinal bleeding.), and gastric reflux (this refers to the abnormal reflux of stomach contents into the esophagus, causing the esophageal lining to be irritated and damaged by stomach acid.) in both groups of patients. Each occurrence of the same symptoms in a single patient was counted as one event. All of the above measures were reported by the treating physician, who was unaware of whether the patient was in the ultrasound group or the control group at the time of reporting.

### 2.4 Statistical methods

Statistical analysis was performed using SPSS 22.0 software. Categorical data are presented as counts, and comparisons between groups were conducted using the chi-square test. A *p*-value < 0.05 was considered statistically significant. Normally distributed variables are described as mean ± standard ( $\bar{x}$ ± s) deviation and analyzed using the independent samples *t*-test (assuming homogeneity of variances); non-normally distributed variables are described as median (IQR) and analyzed using the Wilcoxon signed-rank test (assuming equal distributions). Effect sizes were reported as mean differences or median differences (Hodges-Lehmann estimate), each accompanied by a 95% confidence interval (CI). Binary variables were described as frequencies (%), and comparisons between groups were performed using the Pearson chi-square test (assuming expected frequency ≥5) or Fisher’s exact test (expected frequency <5). Effect sizes were reported as odds ratios (OR) and their 95% CIs (Mantel–Haenszel method). Due to the limited sample size (*n* = 60) and the small number of positive events in this study, no adjustment for multiple confounders was performed; however, baseline characteristics (gender, age, BMI, APACHE II score, etc.) were found to be balanced and comparable between the two groups (*p* > 0.05). All tests were two-sided, with a significance level of *α* = 0.05.

## 3 Results

### 3.1 The general characteristics of the two groups for patients

The gender composition, age, BMI, time of beginning enteral nutrition, nutritional risk score 2002 (NRS 2002), initial acute physiology and chronic health evaluation II (APCHE II) score, sequential organ failure assessment (SOFA) score, and other general information showed no significant statistical differences between the two groups (*p* > 0.05), as presented in Table 1.

### 3.2 GRV assessment operation time, 48 h enteral nutrition compliance rate and enteral nutrition compliance time between the two groups

The ultrasound group demonstrated a significant reduction in both operation time and the time required for enteral nutrition to reach the standard, compared to the control group (*p* < 0.05). However, there was no statistically significant difference in the rate of enteral nutrition reaching the standard between the two groups (*p* > 0.05), as presented in Table 2.

**Table 2:** Comparison of GRV assessment operation time, 48 h enteral nutrition compliance rate and enteral nutrition compliance time between the two groups.

Item	Ultrasound group (*n* = 30)	Control group (*n* = 30)	*Z*/χ^2^	OR (95%CI)	*P*
Time required for nursing care[min, M (Q1, Q3)]	4.6 (2.6, 6.5)	8.2 (5.3, 11.7)	2.138^1^	0.276 (−6.92, −1.60)	0.032
48 h enteral nutrition compliance rate (*n*)			0.111^2^	0.033 (−0.16, 0.22)	0.912
Yes	25	24			
No	5	6			
Time to achieve target enteral nutrition levels [h, M (Q1, Q3)]	34 (26, 39)	42 (28, 46)	6.265^1^	0.809 (−10.44, −5.51)	0.000

1 is the Z-score, and 2 is the χ^2^ value.

### 3.3 VAP and feeding intolerance in the early enteral nutrition process of the two groups of patients

The incidence of diarrhea, constipation, abdominal distension, and aspiration in the ultrasound group was significantly lower than that in the control group during enteral nutrition (*p* < 0.05). There were no significant differences observed between the two groups regarding the occurrence of VAP and gastrointestinal bleeding (*p* > 0.05), as presented in Table 3. It is worth noting that VAP is a major complication among ICU patients, and its occurrence is associated with various factors (such as duration of mechanical ventilation, cuff pressure, oral care, and depth of sedation). Although the incidence of VAP was lower in the intervention group than in the control group in this study (16.7% vs. 20.0%, *p* = 0.739), the difference was not statistically significant. However, the statistical power of the current sample size (30 patients per group) is only 12–15%, meaning that even if the intervention truly reduced the risk of VAP by 20%, this study would still have an 85% or higher probability of concluding “no significant difference” (i.e., an extremely high risk of Type II error). Furthermore, the incidence of VAP is influenced by confounding factors such as mechanical ventilation management strategies. This study did not stratify or adjust for ventilation modes, cuff pressure monitoring frequency, or oral care protocols, which may have further weakened the statistical power of the analysis. Therefore, the results of this study should be interpreted with caution. The current findings do not support the conclusion that “the risk of VAP decreased in the control group,” but this possibility should not be ruled out. The lack of statistical significance in the VAP difference is primarily attributed to insufficient statistical power rather than the ineffectiveness of the intervention. Similarly, the incidence of gastrointestinal bleeding was 6.7% in the intervention group and 16.7% in the control group, with an OR of 0.357 (95% CI: 0.064–2.002) and *p* = 0.228; this negative result may also reflect a Type II error. Although VAP and gastrointestinal bleeding did not reach statistical significance, the incidence rates in the intervention group showed a trend toward clinical benefit (an absolute reduction of 3.3% for VAP and 10.0% for gastrointestinal bleeding). These trends warrant further validation in studies with larger sample sizes.

**Table 3:** Comparison of VAP and feeding intolerance between the two groups.

Item	Ultrasound group (*n* = 30)	Control group (*n* = 30)	OR (95%CI)	*χ* ^2^	*P*
VAP			0.800 (0.217, 2.949)	0.111	0.739
Yes	5 (16.7%)	6 (20.0%)			
No	25 (83.3%)	24 (80.0%)			
Diarrhea (*n*, %)			0.300 (0.092, 0.978)	4.800	0.028
Yes	5 (16.7%)	12 (40.0%)			
No	25 (83.3%)	18 (60.0%)			
Constipation (*n*, %)			0.201 (0.06, 0.72)	6.648	0.010
Yes	4 (13.3%)	13 (43.3%)			
No	26 (86.7%)	17 (56.7%)			
Abdominal distension (*n*, %)			0.286 (0.09, 0.89)	4.800	0.028
Yes	6 (20.0%)	14 (46.7%)			
No	24 (80.0%)	16 (53.3%)			
Gastrointestinal bleeding (*n*, %)			0.357 (0.06, 2.00)	1.456	0.228
Yes	2 (6.7%)	5 (16.7%)			
No	28 (93.3%)	25 (83.3%)			
Reflux (*n*, %)			0.250 (0.08, 0.77)	5.934	0.015
Yes	6 (20.0%)	15 (50.0%)			
No	24 (80.0%)	15 (50.0%)			

## 4 Discussion

### 4.1 Ultrasound monitoring of GRV can reduce nursing time

Previous studies have shown that ultrasound assessment of the GRV is simple to perform and easy to learn; with repeated practice, nurses can achieve a measurement accuracy of over 95% (6). The results of this study indicate that ultrasound assessment of GRV takes less time than the syringe aspiration method. This may be because ultrasound assessment offers high accuracy while requiring fewer steps and less effort, transforming what was once a complex and cumbersome nursing procedure into a simple and straightforward data measurement. This reduces the workload of ICU nurses, consistent with the findings of Brotfain’s study (7) conducted abroad. Ultrasound examinations are painless and well-tolerated by patients; they allow for a direct assessment of antral contraction and can further calculate the antral motility index to evaluate gastric emptying function (8). Research by Pan Hong et al. (9) indicates that using ultrasound to monitor GRV can extend the standard monitoring interval from every 4 h to every 8 h. This monitoring method reduces the frequency of procedures, minimizes interruptions to feeding, cuts down on the time nurses spend at the bedside, and improves work efficiency, thereby playing a positive role in alleviating the workload of critical care nursing.

### 4.2 Ultrasound monitoring of GRV can shorten the time required to achieve target levels of enteral nutrition, but has no effect on the rate of achieving feeding targets

Initiating enteral nutrition early (within 24–48 h) in critically ill patients can protect gastrointestinal structure and function, enhance immune function, and reduce mortality and the incidence of complications (10). According to the literature (11), more than 50% of mechanically ventilated patients with AECOPD are malnourished; early enteral nutrition can reduce the incidence of infections, shorten hospital stays, and lower treatment costs (12). The results of this study show that there was no significant difference in the 48-h enteral nutrition achievement rate between the two groups; however, the enteral nutrition protocol guided by bedside ultrasound assessment of GRV by nurses significantly reduced the time to achieve enteral nutrition targets in mechanically ventilated patients with AECOPD. This may be because the severity of the disease was essentially similar in both groups (no differences in APACHE II or SOFA scores), resulting in similar overall 48-h achievement rates. However, the use of bedside ultrasound by nurses to assess GRV does not interrupt enteral nutrition and avoids the disruption to gastrointestinal function and the digestive process caused by repeated aspiration and re-infusion of gastric fluid, resulting in a shorter time to achieve the target. A study by Cao Lan et al. (13) also demonstrated that bedside ultrasound monitoring of GRV not only reduced the nurses’ workload but also shortened the time required for patients to achieve target feeding volumes. A related meta-analysis (14) further indicated that patients whose enteral nutrition was guided by ultrasound-assessed GRV achieved feeding targets earlier compared to those using traditional gastric aspiration methods.

### 4.3 Ultrasound monitoring of GRV can reduce the incidence of complications

Early enteral nutritional support can significantly reduce mortality and the incidence of complications in critically ill patients; however, its implementation is also associated with risks such as reflux, aspiration, abdominal distension, diarrhea, feeding intolerance, and even intestinal ischemia and necrosis, and may increase the incidence of adverse outcomes (15, 16). In this study, we found that using ultrasound-guided GRV assessment to guide early enteral nutrition resulted in lower rates of constipation, abdominal distension, and gastric reflux in patients, with no difference in the incidence of diarrhea or gastrointestinal bleeding between the two groups. This may be because, during the gastric aspiration method, factors such as patient position and nasogastric tube placement can cause the tube to adhere to the gastric wall or blockage at the tube tip, resulting in incomplete aspiration and consequently lower GRV measurements; the aspirated fluid must be reinfused into the stomach, passively increasing the EN flow rate, which in turn increases the risk of feeding intolerance and reflux. Previous literature has reported that re-injecting aspirated gastric fluid into the patient’s stomach is equivalent to passively pulsing the rate and total volume of enteral feeding, which may increase the risk of enteral nutrition complications (17). In contrast, assessing GRV via ultrasound helps ICU nurses better understand the patient’s gastrointestinal function and, based on this, implement individualized enteral nutrition that better aligns with the patient’s pathophysiological changes.

## 5 Conclusion

In summary, nurse-led bedside ultrasound-based GRV monitoring plays a significant role in the early implementation of enteral nutrition for patients with acute exacerbations of chronic obstructive pulmonary disease (AECOPD) who are on mechanical ventilation. It can reduce the nursing workload and help patients achieve enteral nutrition targets sooner.

## 6 Limitations

This study has the following limitations: ① The sample size was small (30 patients per group), resulting in insufficient statistical power to detect outcomes with low incidence rates; negative results may represent Type II errors; ② The study population consisted of ICU patients; the GRV monitoring protocol may not be applicable to general wards or pediatric patients. Furthermore, as the study was conducted at a single center, patient characteristics, healthcare provider experience, and nutritional support protocols may differ from those at other institutions, limiting external validity; caution is warranted when generalizing the results; ③ Operators were not blinded, which may introduce performance bias; however, data analysts remained blinded to mitigate bias; ④ Other potential sources of bias include measurement bias (consistency of ultrasound procedures was not assessed) and unmeasured confounding factors. ⑤ This study was not registered prior to enrolling the first participant, which may affect the transparency and reproducibility of the results. Although we strictly adhered to the CONSORT guidelines in our reporting, we recommend that readers take this limitation into account when interpreting the results. These limitations suggest that the findings of this study are exploratory in nature and require validation through large-scale, multicenter studies; furthermore, future studies should be registered in advance.

## Data Availability

The raw data supporting the conclusions of this article will be made available by the authors, without undue reservation.
